# Delayed Anticoagulation-Related Intracranial Haemorrhage after Minor Head Injury

**DOI:** 10.1155/2013/412931

**Published:** 2013-12-04

**Authors:** Christopher Beynon, Berk Orakcioglu, Harald Winkler, Nicolas A. Geis, Andreas W. Unterberg, Oliver W. Sakowitz

**Affiliations:** ^1^Department of Neurosurgery, Heidelberg University Hospital, Im Neuenheimer Feld 400, 69120 Heidelberg, Germany; ^2^Department of Anaesthesiology, Heidelberg University Hospital, Im Neuenheimer Feld 110, 69120 Heidelberg, Germany; ^3^Department of Cardiology, Heidelberg University Hospital, Im Neuenheimer Feld 410, 69120 Heidelberg, Germany

## Abstract

Treatment with anticoagulants and antiplatelet agents are well-known risk factors for an unfavourable outcome after traumatic brain injury (TBI). Guidelines for decision making in patients who sustained mild head injury do not apply to anticoagulated patients and therefore, in these cases diagnostic and therapeutic procedures have to be tailored patient-specific. A 69-year-old patient was referred to our hospital after sustaining mild head injury. Due to anticoagulation therapy, a cranial computed tomography was carried out and was without pathologic findings. After negative workup for TBI, the patient was admitted to the ward solely because of intermittent cardiac arrhythmia. The next day, the patient developed a hemiparesis and repeated brain imaging showed a large posttraumatic intracranial haematoma which had to be evacuated surgically. In the further clinical course, the patient recovered completely and a cardiac pacemaker was implanted. Emergency physicians have to be highly alert with anticoagulated patients after head injury, even if the trauma was mild and initial diagnostic procedures demonstrate no acute pathology. Delayed traumatic intracranial haemorrhage may have fatal consequences for patients and while the threshold for admission to a hospital ward should be low, adequate observation at home has to be ensured if patients are discharged.

## 1. Introduction

Increased age and treatment with oral anticoagulants are among the most relevant risk factors for an unfavourable outcome after traumatic brain injury (TBI) [1]. In clinical practice, the majority of TBI patients present to the emergency department after mild TBI in a good clinical condition. Guidelines are available for the treatment of patients with mild TBI [2]; however, in cases of preinjury use of oral anticoagulants, specific measures have to be carried out. We report the case of an anticoagulated patient with mild TBI who would have been discharged home from a neurosurgical standpoint. Routine ECG monitoring revealed severe sinus bradycardia which was the only reason to prompt admission to a cardiac ward for observation and work-up. Twenty hours after admission, the patient developed delayed traumatic intracranial haemorrhage (DTICH) requiring surgical haematoma evacuation.

## 2. Case Report

**Figure 1:**
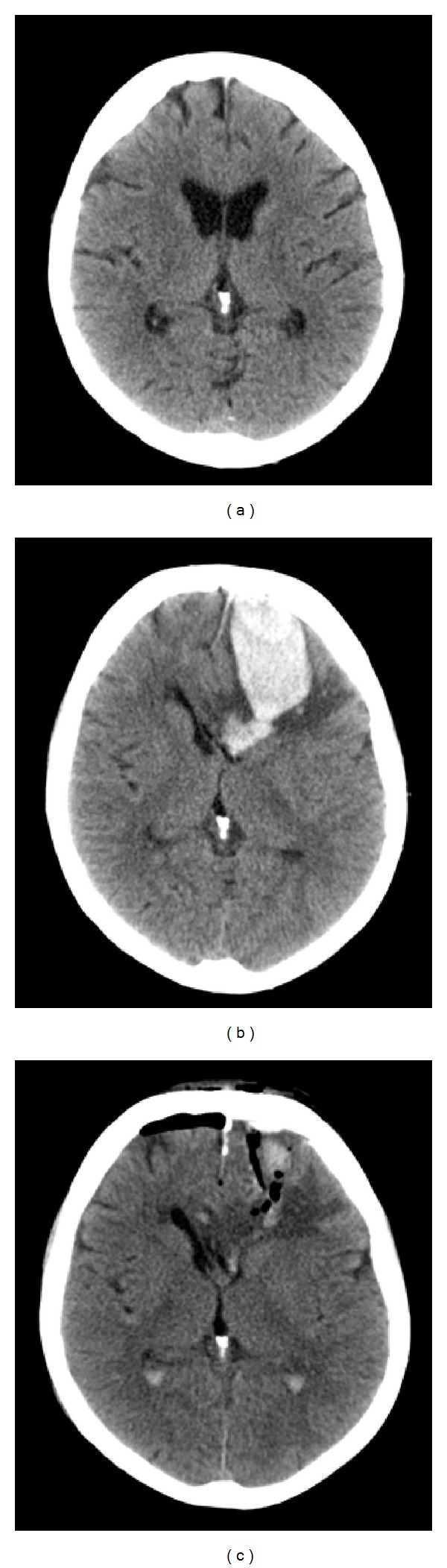
CT images performed initially after the head injury (a), after neurological deterioration (b), and after surgical haematoma evacuation (c).

A 69-year-old woman was brought to our hospital after repeatedly collapsing while going for a walk with a friend. The patient reported that she had fallen on her head but did not complain of headache. On clinical examination the patient was alert, had no focal neurologic deficits, and did not show any signs of traumatic injury. She was on continuous oral anticoagulation therapy with phenprocoumon for prevention of thrombus formation since surgical implantation of artificial aortic and mitral heart valves ten years ago. Blood test results revealed a therapeutic international normalized ratio (INR) of 2.4 with other laboratory values in normal ranges. A cranial computed tomography (CT) scan was performed and showed no pathological findings (Figure 1(a)). Despite negative work-up for TBI, we admitted the patient to the cardiac ward because continuous ECG monitoring showed intermittent episodes of sinus bradycardia below 50 beats per minute. Twenty hours after admission, the patient complained of severe headache and vomited repetitively. The neurological condition had not been determined on a regular basis up to this point. Nevertheless, the patient had been alert and had not complained for headaches five hours before deterioration was discovered. Neurologic examination was remarkable for decreased vigilance (Glasgow Coma Scale (GCS) score 11) and a right-sided hemiparesis. We performed an urgent CT scan which showed an intracerebral haematoma in the left frontal lobe with a total volume of 70 mL and space-occupying mass effect on surrounding brain tissue (Figure 1(b)). The patient underwent urgent surgery and the haematoma was removed in endoscopic technique after burr-hole trepanation. Postoperatively, a CT scan showed near-complete removal of the haematoma (Figure 1(c)). The patient recovered well without neurologic deficits and was transferred to a rehabilitation hospital on the 10th postoperative day. In her further clinical course, a cardiac pacemaker was implanted after detailed cardiologic assessment revealed the diagnosis of sick sinus syndrome.

## 3. Discussion

Hospital admission due to head injury is relatively common and of respective patients, approximately 90% have sustained mild TBI and present to emergency departments with initial GCS scores of 13 to 15 [3]. In industrialized countries especially oral anticoagulants are increasingly prescribed due to the high incidence of cardiovascular diseases. It is well recognized that anticoagulated patients are at increased risk due to their increased bleeding tendency if they sustain TBI [1]. Standard diagnosis and treatment algorithms for patients who sustained mild TBI are only partially applicable to anticoagulated patients [2]. Treatment modalities for anticoagulated patients with mild TBI have been subject of much debate in the last decade, and evidence-based guidelines are not available. The grade of anticoagulation correlates with the incidence of posttraumatic intracranial haemorrhage [4], and therefore laboratory examination of INR should be carried out in order to exclude supratherapeutic levels of anticoagulation. CT imaging should be considered in anticoagulated patients even in the absence of neurological deficits or without loss of consciousness. A retrospective review of seven studies demonstrated that 18% of anticoagulated patients with mild TBI had intracranial haemorrhage on their initial CT scan [5]. However, even a normal CT scan does not rule out development of DTICH as our reported case demonstrates, and this may have serious consequences.

The phenomenon of DTICH has been described as early as 1891 by the German pathologist Otto Bollinger. “*Traumatische Spät-Apoplexie*” was the term for his findings on four patients who sustained a mild head injury and died days to weeks later after they deteriorated neurologically. DTICH has been described in TBI patients without intake of anticoagulation medication, but available studies suggest that there is a close correlation between incidence of intracranial haemorrhage and disorders of the haemostatic system [6]. Two prospective studies on 224 anticoagulated patients with mild TBI demonstrated that 4% of patients with unremarkable initial CT scan had intracranial haemorrhage in follow-up scans after 20 to 24 hours [5]. Cohen et al. [7] retrospectively reviewed their trauma databases and identified 77 patients with mild TBI and concurrent warfarin therapy. Of all patients discharged home after assessment in the emergency department, 18 patients returned with significant traumatic intracranial haemorrhage. Two patients died at home with an autopsy examination of one patient showing an acute subdural haematoma as the cause of death. The reported mortality rate was above 80%, and considering that all patients had been in a good clinical condition at their initial presentation, this very high mortality rate should remind all care-takers and physicians to be highly alert with anticoagulated patients that have sustained mild TBI even if initial testing is unremarkable. In the further course, specific focus should be drawn on the development of neurologic abnormalities or decrease in vigilance which may be indicative signs for DTICH. Especially in patients with increased risks for DTICH (e.g., supratherapeutic anticoagulation), admission to surveillance unit with staff trained in neurological assessment should be considered. In these cases, vigilance should be monitored by regular GCS assessment.

## 4. Conclusion

In our opinion, it is reasonable to discharge patients on anticoagulation therapy who sustained mild TBI if a CT scan is without acute intracranial pathology and sufficient observation at home is assured. Laboratory examination should be carried out in every anticoagulated head-injured patient in order to rule out excess anticoagulation. Additionally, it is crucial to give appropriate instructions to patients and relatives regarding identification of neurological deterioration. If in doubt, the threshold for admission to a hospital ward for observation should be low.
